# Pre-diagnostic blood biomarkers for adult glioma

**DOI:** 10.3389/fonc.2023.1163289

**Published:** 2023-05-17

**Authors:** Lily J. Andrews, Philippa Davies, Christopher Herbert, Kathreena M. Kurian

**Affiliations:** ^1^ Medical Research Council (MRC) Integrative Epidemiology Unit (IEU), Bristol Medical School, University of Bristol, Bristol, United Kingdom; ^2^ Population Health Sciences, Bristol Medical School, University of Bristol, Bristol, United Kingdom; ^3^ Cancer Research Integrative Cancer Epidemiology Programme, University of Bristol, Bristol, United Kingdom; ^4^ Bristol Haematology and Oncology Centre, University Hospitals Bristol National Health Service (NHS) Foundation Trust, Bristol, United Kingdom; ^5^ Brain Tumour Research Centre, Bristol Medical School, University of Bristol, Bristol, United Kingdom

**Keywords:** glioblastoma, glioma, liquid biomarkers, pre-diagnostic, early detection

## Abstract

Glioma is one of the most common malignant primary brain tumours in adults, of which, glioblastoma is the most prevalent and malignant entity. Glioma is often diagnosed at a later stage of disease progression, which means it is associated with significant mortality and morbidity. Therefore, there is a need for earlier diagnosis of these tumours, which would require sensitive and specific biomarkers. These biomarkers could better predict glioma onset to improve diagnosis and therapeutic options for patients. While liquid biopsies could provide a cheap and non-invasive test to improve the earlier detection of glioma, there is little known on pre-diagnostic biomarkers which predate disease detection. In this review, we examine the evidence in the literature for pre-diagnostic biomarkers in glioma, including metabolomics and proteomics. We also consider the limitations of these approaches and future research directions of pre-diagnostic biomarkers for glioma.

## Introduction

Glioma is the most common (~75%) group of primary brain tumours in adults with an overall survival of less than 20% over 5 years, and therefore there is a need to better identify biomarkers for prediction and to support early diagnosis (1). Diagnosis can be difficult as many glioma patients present with non-specific symptoms such as headaches (2). Currently the gold standard test for identifying glioma is magnetic resonance imaging (MRI) and computerized tomography (CT) scans, however these tests cause significant burden to the NHS in terms of cost and long wait times (2). Liquid biopsies such as a blood test could be used as a tool to triage patients for further scans (2). Moreover, a liquid biopsy could have utility for disease monitoring and prediction of disease recurrence (3).

Previous literature has identified putative predictive factors which are associated with glioma, some of these factors include atopic disease and diabetes (4–6). A number of nested case-control studies have attempted to define pre-diagnostic biomarkers for glioma; some of which are informed by putative factors and others carry out a hypothesis-free investigation. However, in some instances it can be difficult to be establish whether the pre-diagnostic biomarker truly predates the onset of glioma or if it represents a change seen earlier in the natural history of disease. In this review we consider any pre-diagnostic biomarker, including proteins and metabolites, which is identified before glioma diagnosis.

## Proteomics

**Figure 1:**
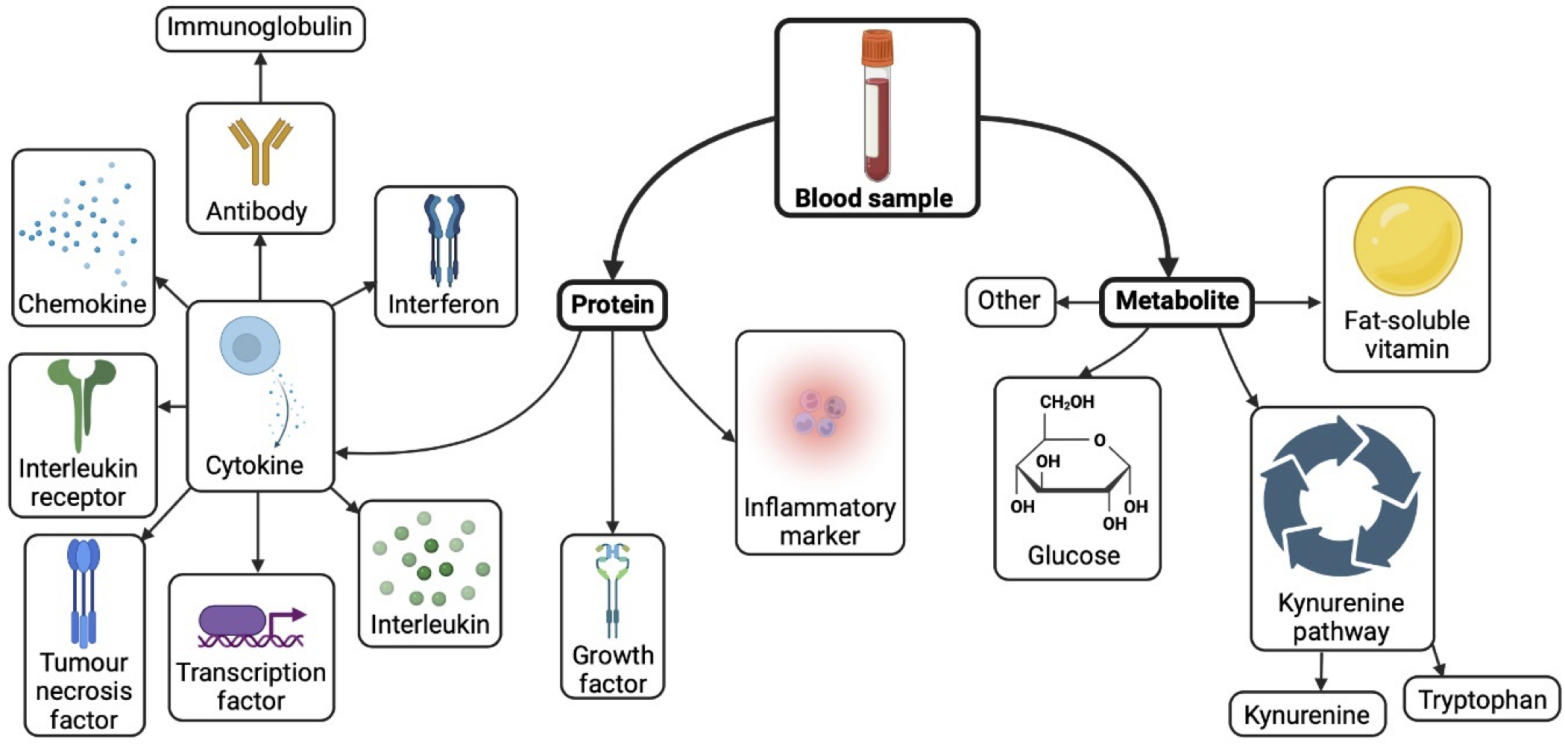
Biomarkers investigated in pre-diagnostic blood of glioma patients. Created with BioRender.com.

Proteomics characterise the global protein landscape within liquid biopsies. Protein alterations linked to cancer could provide novel biomarkers for monitoring disease and early detection of cancer (7). Studies measuring protein marker levels in pre-diagnostic glioma patients compared to control individuals could inform pre-diagnostic biomarker discovery (**Figure 1**). A number of pre-diagnostic protein biomarkers have been suggested to associate with glioma, these are outlined and considered below (**Table 1**).

**Table 1:** Pre-diagnostic biomarkers associated (p-value < 0.05) with glioma risk at any time point against controls.

Author	Liquid biopsy	Glioma subtype	Cohort	Number of samples	Biomarker	Odds Ratio (95% confidence interval)	P-value
Brenner et al. (8)	Serum - proteins	Glioma	Department of Defense Serum Repository	457 cases and 457 controls	IL15	NA	0.002
					IL16	NA	0.001
Lönn et al. (9)	Serum - proteins	Glioblastoma n=12 (54.5%) Gliomas n=4 (18.2%) Unspecified malignant intracerebral tumours n=6 (27.3%)	Alpha-Tocopherol, Beta-Carotene Cancer Prevention Study	22 cases and 400 controls	IGF-I/IGFBP-3 molar ratio	NA	0.03
Späth et al. (10)	Serum - proteins	Glioblastoma	Janus Serum Bank	396 cases and 590 controls	EGFR	1.58 (1.13-2.22)	0.008
		Glioblastoma n=396 (66.8%) Oligodendroglioma n=33 (5.6%) Ependymoma n=27 (4.5%) Astrocytoma n=94 (15.9%) Other subtypes n=43 (7.2%)		593 cases and 590 controls	HER2	1.39 (1.00-1.93)	0.049
		Glioblastoma		396 cases and 590 controls	HER2	1.63 (1.09-2.44)	0.017
Schwartzbaum et al. (11)	Serum - proteins	Glioblastoma	Janus Serum Bank	315 cases and 315 controls	TGFB2	0.87 (0.76-0.98)	<0.05
Schwartzbaum et al. (12)	Serum - proteins	Gliomas grade 1-3 n=172 (35.3%) Glioblastoma n=315 (64.7%)	Janus Serum Bank	487 cases and 487 controls	sIL10RB	0.69 (0.55-0.87)	<0.05
					VEGF	1.46 (1.18-1.82)	<0.05
					IL4	1.13 (0.90-1.43)	<0.05
					sIL4RA	0.92 (0.76-1.12)	<0.05
					IL4-sIL4RA	1.37 (1.16-1.61)	<0.05
Wu et al. (13)	Plasma - proteins	Glioblastoma n=81 (61.8%) Glioma malignant n=3 (2.3%) Glioma mixed astrocytoma n=1 (0.8%) Astrocytoma grades I-II n=9 (6.9%) Astrocytoma anaplastic grade III n=17 (13%) Pilocytic astrocytoma n=4 (3.1%) Pleomorphic xanthoastrocytoma n=2 (1.5%) Oligodendroglioma n=9 (6.9%) Oligodendroglioma anaplastic n=5 (3.8%)	Northern Sweden Health and Disease Study	133 glioma cases and 133 controls	sIL2Rα	1.48 (1.01-2.16)	0.044
					sIL6R	1.90 (1.14-3.17)	0.014
					sTNFR2	1.72 (1.01-2.93)	0.045
					sVEGFR2	2.44 (1.29-4.61)	0.006
Björkblom et al. (14)	Serum - metabolites	Glioblastoma	Janus Serum Bank	110 cases and 110 controls	γ-tocopherol	2.1 (1.2-3.8)	0.0009
					α-tocopherol	1.7 (1.0-3.0)	0.0018
					2-keto-L-gluconic acid	NA	0.007
					Erythritol	NA	0.0221
					N-acetyl-L-alanine	NA	0.0314
					Xylose	NA	0.0386
					Erythronic acid	NA	0.0391
Huang et al. (15)	Serum - metabolites	High grade glioma n=41 (64.1%) Lower-grade n=19 (29.7%) Unknown grade n=4 (6.25%)	Alpha-Tocopherol, Beta-Carotene Cancer Prevention Study	64 cases and 64 controls	Glutamate	0.65 (0.43-0.96)	0.0321
					N-Acetylleucine	0.67 (0.44-1.00)	0.0499
					Cysteine	0.39 (0.19-0.77)	0.0069
					Cysteine-S-sulfate	0.62 (0.40-0.96)	0.0323
					N-Acetyltyrosine	0.57 (0.36-0.88)	0.0109
					N-Acetylphenylalanine	0.63 (0.41-0.96)	0.0326
					Phenyllactate	0.67 (0.46-0.97)	0.0350
					Tyrosine	0.71 (0.50-1.00)	0.0477
					N-Acetylkynurenine	0.65 (0.43-0.98)	0.0420
					N-Acetyltryptophan	0.64 (0.41-0.98)	0.0421
					Xanthurenate	0.67 (0.45-0.99)	0.0453
					2-Oxoarginine	0.56 (0.37-0.85)	0.0065
					Argininate	0.60 (0.42-0.88)	0.0083
					N-Acetylarginine	0.69 (0.48-0.98)	0.0407
					Mannitol/sorbitol	0.63 (0.41-0.97)	0.0374
					Pyruvate	0.65 (0.43-0.97)	0.0343
					Trigonelline	1.72 (1.10-2.69)	0.0182
					Alpha-tocopherol	0.65 (0.44-0.96)	0.0305
					Alpha-ketoglutarate	0.52 (0.32-0.84)	0.0075
					Stearoylcaritine	1.58 (1.09-2.29)	0.0159
					Margaroylcarnitine	1.50 (1.05-2.15)	0.0251
					Eicosenoylcarnitine	1.59 (1.01-2.51)	0.0457
					1-Palmitoyl-2-linoleoyl-GPI	0.61 (0.41-0.91)	0.0147
					Glycerophosphorylcholine	1.76 (1.00-3.10)	0.0484
					1-(1-Enyl-palmitoyl)-2-oleoyl-GPC	1.47 (1.01-2.15)	0.0449
					Chenodeoxycholate	0.56 (0.37-0.86)	0.0082
					Cholate	0.60 (0.39-0.91)	0.0162
					3β-Hydroxy-5-cholenoic acid	0.67 (0.45-0.98)	0.0393
					Glycocholenate sulfate	0.64 (0.41-0.98)	0.0420
					Sphingomyelin	1.67 (1.07-2.61)	0.0228
					Cytidine	1.49 (1.01-2.19)	0.0471
					Gamma-glutamyltyrosine	0.61 (0.40-0.92)	0.0175
					Propyl 4-hydroxybenzoate	0.54 (0.32-0.92)	0.0230
					Methyl 4-hydroxybenzoate sulfate	0.67 (0.46-0.97)	0.0320
					3-Methyl catechol sulfate	1.53 (1.02-2.28)	0.0380
					Quinate	1.52 (1.03-2.25)	0.0334
					1-Methylurate	1.58 (1.08-2.30)	0.0171
					1-Methylxanthine	1.63 (1.09-2.46)	0.0184
					Paraxanthine	1.52 (2.05-2.22)	0.0284
					Theobromine	1.53 (1.02-2.28)	0.0375
					5-Acetylamino-6-amino-3-methyluracil	1.55 (1.02-2.35)	0.0379
					Theophylline	1.50 (1.02-2.22)	0.0412
					7-Methylxanthine	1.47 (1.01-2.14)	0.0415
	Serum - metabolites	High-grade glioma	Alpha-Tocopherol, Beta-Carotene Cancer Prevention Study	41 cases and 41 controls	N-Acetylglutamate	1.99 (1.10-3.61)	0.0228
					2,3-Dihydroxy-2-methylbutyrate	2.05 (1.09-3.85)	0.0258
					Cysteine	0.44 (0.20-0.98)	0.0437
					Ribonate	2.53 (1.02-6.25)	0.0445
					Oxalate	0.55 (0.33-0.91)	0.0211
					Threonate	0.57 (0.34-0.95)	0.0300
					Gulonate	1.99 (1.04-3.81)	0.0387
					Oleoyl-oleoyl-glycerol	1.85 (1.03-3.31)	0.0402
					Cholate	0.54 (0.31-0.93)	0.0273
					Glycocholenate sulfate	0.39 (0.19-0.79)	0.0091
					3β-Hydroxy-5-cholenoic acid	0.49 (0.28-0.85	0.0106
					5Alpha-pregnan-3beta, 20beta-diol monosulfate	0.56 (0.34-0.92)	0.0209
					Pregnenolone	0.53 (0.30-0.94)	0.0293
					5-Methyluridine	2.25 (1.12-4.52)	0.0226
					Tartronate	0.57 (0.34-0.97)	0.0383
					Methyl 4-hydroxybenzoate sulfate	0.47 (0.26-0.83)	0.0097
					Propyl 4-hydroxybenzoate sulfate	0.52 (0.28-0.97)	0.0391
Jonsson et al. (16)	Plasma - metabolites	Glioblastoma n=43 (67.2%) Oligodendroglioma n=6 (9.4%) Anaplastic oligodendroglioma n=2 (3.1%) Astrocytoma, anaplastic type n=6 (9.4%) Astrocytoma n=2 (3.1%) Glioma not specified n=5 (7.8%)	Northern Sweden Health and Disease Study	64 cases and 64 controls	myo-Inositol	NA	0.0002
					scyllo-Inositol	NA	0.047
					Cysteine	NA	0.014
					Glycine	NA	0.0044
					Glyceric acid	NA	0.026
					Aceturic acid	NA	0.0083
					Phosphate	NA	0.014
					Proline	NA	0.022
					4-Hydroxyphenylacetic acid	NA	0.032
					Erythronic acid	NA	0.0043
					Erythritol	NA	0.013
					N-acetylglucosamine	NA	0.016
					Creatinine	NA	0.025
					Uric acid	NA	0.046
					Urea	NA	0.039
Schwartzbaum et al. (17)	Serum - metabolites	High grade glioma n=476 (78.8%) Other glioma: n=128 (21.2%) Other glioma n=44 (21.2%)	Apolipoprotein mortality risk	604 cases and 527,976 controls	Glucose	NA	0.002
		High grade glioma n=164 (78.8%) Other glioma n=44 (21.2%)	Metabolic syndrome and Cancer project	208 cases and 269,157 controls	Glucose	NA	0.04

NA, not available.

## Growth factors

Epidermal growth factor receptor (*EGFR*) is an oncogene implicated in glioma initiation, tumour growth and progression (18, 19). Späth et al. measured pre-diagnostic serum levels of EGFR and human epidermal growth factor receptor 2 (HER2) in glioma patients. EGFR (Odds Ratio (OR)=1.58, 95% confidence interval (95% CI)=1.13-2.22) and HER2 (OR=1.63, 95% CI=1.09-2.44) were found to be elevated in serum samples with an increased risk of glioblastoma development. However only elevated HER2 levels (OR=1.39, 95% CI=1.00-1.93) were found to be associated with increased glioma risk (10). Insulin-like growth factor (IGF-1) has similar downstream signaling pathways to EGF (20). Some studies found no evidence of insulin-like growth factor 1 (IGF-I), insulin-like growth factor binding protein 1 (IGFBP1), IGFBP2, IGFBP-3, or IGF-I/IGFBP3 ratio being associated with glioma risk (9, 12, 21, 22). However, Lönn et al. found conflicting evidence with IGF-I/IGFBP-3 ratio found to be associated with glioma risk, although the sample size was small (9).

Schwartzbaum and colleagues investigated other growth factors from different pathways, with transforming growth factor-beta 2 (TGFB2) found to be inversely related to glioblastoma (OR=0.87, 95% CI=0.76-0.98) in the Janus Serum Bank (JSB) (11). Although, this relationship was not identified in all glioma cases as evidenced in other studies (11, 12). Increased circulating vascular endothelial growth factor (VEGF) was found to be associated with pre-diagnostic glioma (OR=1.46, 95% CI=1.18-1.82) in the JSB, whilst two other studies did not find the same relationship within the Department of Defense Serum Repository (DoDSR) and Northern Sweden and Disease Study cohort (NSHDS) cohorts (8, 12, 13). Increased levels of soluble vascular endothelial growth factor receptor 2 (sVEGFR2) was found to be associated with increased glioma risk (OR=2.44, 95% CI=1.29-4.61) (13). No associations were found between placental growth factor (PLGF), hepatocyte growth factor (HGF), transforming growth factor beta 1 (TGFβ1), fibroblast growth factor 2 (FGF2) and transforming growth factor alpha (TGFα) levels and pre-diagnostic glioma patients (8, 12, 13).

## Immunoglobulin

Many epidemiological studies have suggested that allergic and atopic conditions have an inverse relationship with glioma risk (5, 23). Most of these papers examine the association between immunoglobulin E (IgE) antibodies which have a role in type I immediate allergic response (24). Schlehofer et al. investigated atopic status through IgE levels in pre-diagnostic glioma cases’ serum versus controls in the European Prospective Investigation into Cancer and Nutrition. High-grade gliomas were found to have a borderline association with IgE levels, with the greater intensity of the IgE, the lower the OR. Interestingly, only women were found to have an inverse association between allergic sensitization (via IgE levels) and all glioma risk, this association was stronger in high-grade gliomas (25). Schwartzbaum et al. identified the same pronounced association between elevated levels of IgE and glioma risk also observed within women in the JSB. This study also observed an association between testing positive for total IgE and a decreased risk of glioma at least 20 years before diagnosis (26). Conversely, Calboli and colleagues did not find an association between elevated IgE levels and risk of glioma, but there was suggestive evidence of an association in the Nurses’ Health Study, Women’s Health Study (WHS), HPFS, and Physicians’ Health Study (PHS) cohort (27). A receptor for IgE, soluble cluster of differentiation 23 (sCD23), was also investigated but no relationship was found with pre-diagnostic glioma in the JSB and NSHDS cohort (12, 13).

Previous literature has postulated an inverse relationship between immune response derived from a virus. These include a history of chickenpox, shingles, and herpes virus and glioma risk (28–30). Sjöström et al. examined immunoglobulin G (IgG) antibodies for cytomegalovirus, varicella-zoster virus (VZV), adenovirus, and Epstein-Barr virus in the NSHDS, Malmö Diet and Cancer Study (MDCS), and the Diet, Cancer, and Health cohort from Copenhagen. While no overall associations were discovered between IgG levels for the viruses and pre-diagnostic glioma, an inverse relationship was found between VZV IgG levels and pre-diagnostic glioma more than two years before diagnosis, and also an inverse association between positive VZV IgG levels and glioblastoma risk (31). However, Brenner et al. identified no relationship between glioma and prior immune-related conditions (this included any allergy, autoimmune disease or a combination) (8).

## Transcription factors

Allergy related transcription factors were investigated to identify potential links in pre-diagnostic disease of glioma in the JSB. Elevated beta-Catenin levels in serum were found to be associated with pre-diagnostic glioma (OR=1.86, 95% CI=1.28-2.71) (12). However, no other associations with pre-diagnostic glioma were found in any of the other allergy related transcription factors that were investigated (forkhead box p3 (FOXP3), signal transducer and activator of transcription 3 (STAT3) and STAT6) (11, 12).

## Interleukins and interleukin receptors

Interleukins (ILs) have key roles in the development, progression, and control of cancer (32). Brenner at al. found increased levels of IL15 and IL16 to be inversely associated with glioma risk in pre-diagnostic individuals in the DoDSR (8). However another study did not find this same association in the JSB (12). Schwartzbaum and colleagues found pre-diagnostic glioma to be associated with increased IL4 levels (OR=1.13, 95% CI=0.90-1.43) in the JSB (12). In their previous paper there was only a suggestive relationship between IL4 and risk of glioblastoma, however this was with decreased levels with IL4 less than or equal to 5 years before diagnosis (11). Studies from Schwartzbaum and Wu et al. also investigated the relationship between other ILs and pre-diagnostic glioma cases; however, these were found not to be associated in the JSB and NSHDS (11–13).

Soluble interleukin receptors have also been investigated to identify their relationship with pre-diagnostic glioma. Schwartzbaum and colleagues found an association with reduced soluble interleukin 4 receptor alpha (sIL4RA) (OR=0.92, 95% CI=0.76-1.12) and elevated IL4-sIL4RA (OR=1.37, 95% CI=1.16-1.61) with increased risk of glioma in the JSB (12). This evidence is also suggested in their previous paper using the same cohort with sIL4RA (OR=0.80, 95% CI=0.65-1.00) and IL4-sIL4RA (OR=1.02, 95% CI=1.01-1.04) (11). Elevated levels of sIL2RA (OR=1.48, 95% CI=1.01-2.16) and sIL6R (OR=1.90, 95% CI=1.14-3.17) was suggested to be associated with glioma risk in the NSHDS, but this relationship was not significant after multiple testing and was not consistent in the JSB (12, 13). Decreased levels of sIL10RB (OR=0.69, 95% CI=0.55-0.87) and Leukemia inhibitory factor (LIF) (OR=0.47, 95% CI=0.23-0.94) was associated with glioma 5 or less years before diagnosis (12). SIL13RA2 and IL12p40 were found to have no relationship with glioma risk in two separate cohorts (8, 11, 12).

## Chemokines

Chemokines are small, secreted proteins that make up one of the largest families of cytokines, and have critical roles in immune function (33). Schwartzbaum et al. identified the only pre-diagnostic chemokine which was C-C motif chemokine 22 (OR=1.45, 95% CI=1.07-1.96 and associated with glioma risk greater than 15 years before diagnosis) in the JSB (12). The other pre-diagnostic chemokines analysed showed no association with glioma, these included monocyte chemoattractant protein 1, thymus and activation regulated chemokine, these were assessed in the JSB or DoDSR cohorts (8, 12). Similarly, monocyte chemoattractant protein 3, macrophage inflammatory protein 1 alpha and beta, fractalkine and chemokine (C-X-C motif) ligand 13 were assessed in JSB and NSHDS with no association (12, 13).

## Interferons

Interferons (IFNs) are a type of cytokine which have been implicated in cancer progression (34). However, to date no association has been found with pre-diagnostic glioma. Brenner and Schwartzbaum et al. investigated interferons in the DoDSR and JSB. Overall, IFN gamma, IFN beta and IFN-alpha/beta receptors were found to have no relationship with glioma risk (8, 11, 12).

## Tumor necrosis factors

Tumour necrosis factors (TNFs) are key regulators in immune and inflammatory response to cancer (35). Only tumour necrosis factor alpha (TNF-α) and TNF receptors have been explored pre-diagnostic bloods of glioma patients. However, no relationship was identified between TNF-α and glioma risk in the JSB and DoDSR (8, 12, 13). Interestingly, while no relationship was found between TNF-α, Schwartzbaum et al. investigated two soluble receptors, soluble tumour necrosis factor receptor 2 (sTNFR2) was found to have an association with glioma risk (OR=1.72, 95% CI=1.01-2.93) but there was no association with sTNFR1 (13). Two soluble molecules in the tumour necrosis receptor family were also studied, sCD27 and sCD30, but no relationship was found with pre-diagnostic glioma in the JSB or NSHDS cohort (12, 13).

## Chronic inflammation

Chronic inflammation is well-established driver of carcinogenesis, it can lead to tumour progression and aid metastasis (36, 37). There are multiple biomarkers which signal systemic inflammation, this is usually due to injury or stress in an individual. These include C-reactive protein (CRP), white blood cell count (WBC) and neutrophil to lymphocyte count (NLR) (38–40). Cote and colleagues published the only study, to our knowledge, looking at biomarkers of inflammation in pre-diagnostic glioma. Data obtained from the UK Biobank cohort suggested a borderline relationship between NLR (hazard ratio=1.54, 95% CI=1.00-2.39, p-trend=0.05) and pre-diagnostic glioma. No other relationship was identified between WBC or CRP and glioma risk (22).

## Metabolomics

Metabolomics is a tool which measures the broad landscape of metabolites within biological samples such as blood, urine, and saliva, and may be detected using techniques such as mass spectrometry and nuclear magnetic resonance (41). An understanding of the pre-diagnostic metabolic signature in liquid biopsies prior to development of cancer compared to control cases could provide insights to novel candidate pre-diagnostic biomarkers. Several pre-diagnostic metabolite markers have been postulated to associate with glioma, which are summarized and discussed below (**Figure 1**, **Table 1**).

## Glucose

Previous studies have suggested an inverse relationship between diabetes and glioma risk (6). Schwartzbaum et al. identified a similar inverse association between pre-diagnostic diabetes and glioma risk in the Apolipoprotein-related Mortality Risk (AMORIS) cohort. The same study identified glucose levels in serum and plasma of pre-diagnostic individuals to have an inverse association with glioma risk within the AMORIS and Metabolic Syndrome and Cancer project (Me-Can) cohorts (17). In contrast, Björkblom and colleagues did not find the same association between glucose levels and pre-diagnostic glioblastoma in the JSB (14).

## Fat-soluble vitamins

Deficiencies in vitamin A isoforms (including retinol, retinoic acid and retinal) and vitamin D (most abundant form in the blood is 25-hydroxyvitamin D (25(OH)D)) have been evidenced to increase the risk of cancer in individuals. Whereas vitamin E, which comprises of two groups, tocopherols and tocotrienols each with four isomers (α, β, γ and δ) is considered to have an inverse role in cancer prevention (42). A number of vitamin isoforms (25(OH)D, retinol, α-tocopherol, and γ-tocopherol) were assessed in pre-diagnostic glioma within UK Biobank, Nurses’ Health Study, and Health Professionals Follow-Up Study (HPFS) by Yue et al. However, there was no evidence of an association in 25(OH)D in serum, and α-tocopherol, γ-tocopherol and retinol in plasma and glioma risk (43). However, Björkblom et al. identified an association in increased levels of α-tocopherol (OR=1.7, 95% CI=1.0-3.0) and γ-tocopherol (OR=2.1, 95% CI=1.2-3.8) related to glioblastoma risk in the JSB (14). Alternatively, Huang et al. reported decreased levels of α-tocopherol (OR=0.65, 95% CI=0.44-0.96) associated with glioma risk in the Alpha-Tocopherol, Beta-Carotene Cancer Prevention (ATBC) study.

## Amino acid metabolism

The amino acid tryptophan is metabolized through the kynurenine pathway in more than 90% of cases and the downstream products of kynurenine pathway have been implicated in carcinogenesis (44). Samanic et al. investigated the levels of tryptophan, kynurenine, and the ratio of kynurenine to tryptophan derived from plasma in pre-diagnostic glioma cases but found no association with circulating plasma in glioma risk within the Nurses’ Health Study and HPFS (45). Similarly, no association was found between tryptophan and glioblastoma risk in the JSB (14).

## Other metabolites

Björkblom et al. investigated 180 small molecular compounds in pre-diagnostic glioblastoma cases compared to matched control individuals in the JSB. This study found elevated levels of γ-tocopherol (OR=2.1, 95% CI=1.2-3.8), α-tocopherol (OR=1.7, 95% CI=1.0-3.0), 2-keto-L-gluconic acid, erythritol, N-acetyl-L-alanine, xylose and erythronic acid to be associated with glioblastoma risk (14). Jonsson et al. carried out a similar study investigating 142 metabolites in a different cohort of pre-diagnostic glioma patients compared to controls in the NSHDS. This study identified elevated plasma levels of myo-inositol, scyllo-inositol, cysteine, glycine, glyceric acid, aceturic acid, phosphate, proline, 4-hydroxyphenylacetic acid, erythronic acid, erythritol, N-acetylglucosamine, creatinine, uric acid and urea to be associated with glioma risk (16). Interestingly, both studies found elevated levels of erythronic acid (P-value: 0.0043 and 0.0391) and erythritol (P-value: 0.013 and 0.0221) related to an increased glioma risk in pre-diagnostic liquid biopsies within the JSB and NSHDS. Huang et al. performed an analysis on 730 known metabolites in pre-diagnostic glioma patients and controls in the ATBC study. In this study a total of 43 metabolites were found to be associated with overall glioma risk before diagnosis. The strongest associations were found between lower levels of 2-oxoarginine (OR=0.56, 95% CI=0.37-0.85) and cysteine (OR=0.39, 95% CI=0.19-0.77) with overall glioma risk and decreased glycocholenate sulfate (OR=0.39, 95% CI=0.19-0.79) and methyl 4-hydroxybenzoate sulfate (OR=0.47, 95% CI=0.26-0.83) with high-grade glioma risk. The other evidenced associations between metabolites and glioma risk have been noted in **Table 1** (15). Notably, in two separate cohorts (ATBC study and NSHDS) cysteine was found to be associated with glioma risk (P-value: 0.0069 and 0.014) (15, 16).

## Limitations of metabolomics and proteomics

The progression of biomarker analysis through multi-omics technologies has allowed novel protein and metabolite biomarkers to be discovered in liquid biopsies (46). Despite common practice of these techniques in biomarker research, there are still some limitations to the use of metabolomics and proteomics platforms.

Firstly, within metabolomics and proteomics there are multiple platforms and assays to detect biomarkers in liquid biopsies. Depending on the method there are different limitations based on sensitivity, reproducibility, or cost of equipment which need to be considered in biomarker analyses (46, 47).

There are some protein isoforms and metabolites which are present in low abundance, so it can be much harder to detect using current technology. The presence of abnormal/malfunctioning proteins in the body tend to be degraded, which means these proteins are also not measured in proteomic analyses (46).

Metabolites and proteins are dynamic molecules which tend to be difficult to detect due to their complex and changing structures. Because of this, it is unfeasible to accurately measure the entire metabolome and metabolite analyses that are carried out require huge amounts of data (46, 48, 49). Protein and metabolite levels can alter in the body day to day, and throughout the life course depending on many factors including age and lifestyle (50). As this review only found publications using a nested case-control study design, most of the liquid biopsies were taken at a single time point only providing a snapshot of the dynamic molecules at that specific time.

## Future directions

In the current literature, metabolomic and proteomic studies investigating pre-diagnostic biomarkers of glioma have only been carried out in the blood (51). Further analysis of other bodily fluids remains an area for future exploration. Some of the likely candidates for pre-diagnostic biomarker discovery in glioma patients include cerebrospinal fluid (CSF) and urine as these liquid biopsies have previously identified potential biomarkers in glioma (52–54). CSF is more likely to show higher levels of central nervous system (CNS) specific biomarkers due to its proximity to the brain, but to obtain the biopsy this requires an invasive procedure which is not routinely performed (54, 55). In contrast, urine is easily accessible and non-invasive, however it may yield much lower levels of CNS specific biomarkers due to proximity, the blood-brain barrier and glomerular filtration (52, 54, 56, 57). Other bodily fluids which could be considered for biomarker discovery include saliva, stool, and breath (58–60). All of these liquid biopsies could be utilized to discover novel pre-diagnostic biomarkers for glioma or to validate markers that have been suggested in this review.

Additionally, there are many other analytes which can be detected in liquid biopsies and utilized in biomarker discovery. These include microRNA, extracellular vesicles, cell-free RNA, circulating tumour cells, and circulating tumour DNA (61–65). However, the potential of these analytes in the early detection of cancer is unknown (66). Some of these suggested analytes may only be detected in a later stage of glioma progression and therefore may not be suitable as pre-diagnostic markers.

## Conclusion

Glioma is a devasting cancer with survival rates less than 20% over 5 years. It is often diagnosed at a late stage of disease progression and usually leads to significant mortality and morbidity. Currently little is known about pre-diagnostic biomarkers which predate glioma detection, but this could improve the earlier detection of glioma. To our knowledge, this review outlines all of the current literature examining studies where biomarkers were assessed pre-diagnosis. Limitations of metabolomics and proteomics in biomarker detection were considered and the future directions for the discovery and validation of pre-diagnostic biomarker for glioma were suggested.

## Author contributions

LA was responsible for article conception, article structure, creating figures and tables, and drafting the manuscript. KK was responsible for drafting the article abstract and providing critical revision of the manuscript. PD and CH provided revisions to the manuscript. All authors contributed to the article and approved of the submitted version.
